# Serum Endostatin Concentrations Are Higher in Men with Symptoms of Intermittent Claudication

**DOI:** 10.1155/2014/298239

**Published:** 2014-01-30

**Authors:** Jonathan Golledge, Paula Clancy, Graeme J. Hankey, Bu B. Yeap, Paul E. Norman

**Affiliations:** ^1^The Vascular Biology Unit, Queensland Research Centre for Peripheral Vascular Disease, School of Medicine and Dentistry, James Cook University, Townsville, QLD 4811, Australia; ^2^Department of Vascular and Endovascular Surgery, The Townsville Hospital, Townsville, QLD 4814, Australia; ^3^School of Medicine and Pharmacology, University of Western Australia, Perth, WA 6907, Australia; ^4^Department of Neurology, Royal Perth Hospital, Perth, WA 6000, Australia; ^5^Department of Endocrinology, Fremantle Hospital, Fremantle, WA 6160, Australia; ^6^School of Surgery, University of Western Australia, Perth, WA 6907, Australia

## Abstract

*Objectives*. A cleavage fragment of collagen XVIII, endostatin, is released into the circulation and has been demonstrated to have antiangiogenic effects in animal models. We hypothesized that circulating endostatin would be increased in patients with symptoms of lower limb peripheral artery disease. *Design*. Cross-sectional study. *Participants*. Community dwelling older men. *Measurements*. Intermittent claudication was defined using the Edinburgh Claudication Questionnaire (ECQ). Serum endostatin was measured by a commercial ELISA. The association of serum endostatin with intermittent claudication was examined using logistic regression adjusting for age, diabetes, hypertension, dyslipidemia, coronary heart disease, and stroke. *Results*. Serum endostatin was measured in 1114 men who completed the ECQ. 106 men had intermittent claudication, 291 had atypical pain, and 717 had no lower limb pain. Mean (±standard deviation) serum endostatin concentrations (ng/mL) were 145.22 ± 106.93 for men with intermittent claudication, 129.11 ± 79.80 for men with atypical pain, and 116.34 ± 66.57 for men with no lower limb pain; *P* < 0.001. A 70 ng/mL increase in endostatin was associated with a 1.17-fold rise in the adjusted odds of having intermittent claudication (OR 1.17, 95% confidence interval 1.00–1.37, and *P* = 0.050). *Conclusions*. Serum endostatin is raised in older men who have symptoms of intermittent claudication. The role of endostatin in the genesis and outcome of peripheral artery disease requires further investigation.

## 1. Introduction

Lower limb ischemia due to atherosclerosis of the peripheral arteries, commonly termed peripheral artery disease, is a common problem in older adults worldwide [1–3]. The symptoms associated with stenosis or occlusion of lower limb arteries are highly variable [4, 5]. A significant proportion of patients with lower limb ischemia (defined as ankle brachial pressure index <0.9) report no leg symptoms [4, 5]. The exact determinants of symptom severity and outcome for patients with lower limb arterial occlusive disease are poorly understood but likely depend on the ability of the limb to adapt to atherosclerosis through development of new capillaries (angiogenesis) and expansion of collateral arteries (arteriogenesis) [6]. Currently, however, the factors determining angiogenesis and arteriogenesis in patients are poorly defined.

Collagen XVIII is a heparan sulphate proteoglycan expressed (amongst other sites) within elastic arteries [7, 8]. A C-terminal breakdown product of collagen XVIII, known as endostatin, has been extensively investigated for its role in suppression of neoplastic tumours [7]. Both animal model and *in vitro* studies suggest that endostatin has anti-angiogenic effects [9–11]. Recombinant endostatin is being investigated as a therapy for a number of neoplastic tumours [12].

Given the likely importance of angiogenesis in determining the symptoms associated with peripheral artery disease we hypothesised that circulating endostatin concentrations would be increased in patients who have intermittent claudication. The aim of this study was to compare serum concentration of endostatin in patients with and without intermittent claudication.

## 2. Methods

### 2.1. Study Design

To examine the association of serum endostatin concentration with symptoms of intermittent claudication we utilized men recruited from the Health in Men Study (HIMS). HIMS was developed from a community based trial of screening for abdominal aortic aneurysm performed between 1996 and 1999 (wave 1) [13]. 4248 men reattended between 2001 and 2004 and completed a second questionnaire and had their blood collected (wave 2). For the current study we randomly selected approximately 25% of men (*n* = 1117) based on previous studies of circulating endostatin concentration which suggested that this number of subjects would provide >80% power to detect a 30% difference between cases and controls [14, 15]. The University of Western Australia Human Research Ethics Committee approved the study, and all men gave written informed consent.

### 2.2. Risk Factors and Medication

Risk factors were defined from questionnaires administered at initial assessment (wave 1) and the time of blood sampling (wave 2). Coronary heart disease (CHD) was recorded in patients with a history of angina, myocardial infarction, or coronary revascularization. Hypertension, diabetes, and dyslipidemia were defined by a past history of diagnosis or treatment for these conditions. Medications recorded at the time of blood sampling including lipid modifying medication, antihypertensive medication, and diabetes medication were used in these definitions. Past history of stroke was also recorded at both assessments and used to define previous stroke.

### 2.3. Definition of Intermittent Claudication

Symptoms of intermittent claudication were defined by responses to the Edinburgh Claudication Questionnaire (ECQ) completed at the time of blood sampling as previously described [15]. The ECQ comprises seven items asking whether pain or discomfort in the legs is present on walking; the distance before pain limits walking; whether pain occurs with standing still or sitting, when walking uphill or hurrying; whether pain occurs with walking at ordinary pace on level ground; and whether pain diminishes within 10 min of resting. A man was classified as positive in the ECQ if he had leg pain when walking, but not sitting or standing, which was relieved by standing still for 10 min. Where pain was present on standing or sitting and not relieved by rest for 10 mins it was defined as atypical.

### 2.4. Measurement of Serum Endostatin

Blood samples were collected between 08:00 h and 10:30 h. Serum was prepared immediately following phlebotomy and stored at −80°C until assayed. Serum endostatin was measured with a commercial assay (R&D Systems, Minneapolis, USA) by an experienced scientist unaware of the case or control status. The intra- and interassay coefficients of variations were 7.3 and 9.0%, respectively.

### 2.5. Statistical Analyses

Quantitative data were not normally distributed (as assessed by Kolmogorov-Smirnov and Shapiro-Wilk tests) and were presented as mean and standard deviation and compared by Mann-Whitney *U* or Kruskal Wallis tests. Nominal data were presented as numbers and percentages and compared by chi-squared test. The association of serum endostatin tertiles (and a 70 ng/mL increase) with intermittent claudication was assessed using multiple regression analysis adjusting for age above 76 years (approximately median age), CHD, hypertension, diabetes, smoking, stroke, and dyslipidemia.

## 3. Results

4,248 men attended for blood sampling and assessment during wave 2 of HIMS. Samples for endostatin measurement were randomly selected on 1117 men of whom 1114 had completed the ECQ. Based on ECQ answers, 106 of these men were defined to have intermittent claudication, 291 had atypical pain (or could not walk), and 717 had no lower limb pain. Mean (±standard deviation) serum endostatin concentrations (ng/mL) were 145.2 ± 106.9 for men with intermittent claudication, 129.1 ± 79.8 for men with atypical pain, and 116.3 ± 66.6 for men with no lower limb pain; *P* < 0.001. Men with atypical pain were excluded from further analysis.

The characteristics of the 106 men with intermittent claudication and the 717 with no leg pain are shown in Table 1. Men with intermittent claudication were older and more likely to have a history of hypertension, diabetes, dyslipidemia, smoking, CHD, and stroke.

The association of serum endostatin with intermittent claudication was assessed by adjusting for age above 76 years, hypertension, diabetes, dyslipidemia, smoking, CHD, and stroke. Serum endostatin concentration was mildly but significantly associated with the presence of intermittent claudication (Table 2). Compared to men with serum endostatin in the first (<93 ng/mL) tertile, men with serum endostatin in the second (93–120 ng/mL) tertile had 2.07 adjusted odds (95% confidence interval 1.15–3.73, *P* = 0.015) of having intermittent claudication, and men with serum endostatin in the third (>120 ng/mL) tertile had 1.78 adjusted odds (95% confidence interval 0.98–3.22, *P* = 0.058) of having intermittent claudication.

In a further analysis we assessed the association of a 70 ng/mL increase in endostatin (approximately one standard deviation) with intermittent claudication after adjusting for age above 76 years, hypertension, diabetes, dyslipidemia, smoking, CHD, and stroke. A 70 ng/mL increase in endostatin was associated with a 1.17-fold rise in the adjusted odds of having intermittent claudication (odds ratio 1.17, 95% confidence interval 1.00–1.37, and *P* = 0.050).

## 4. Discussion

The main finding of this study was that serum concentrations of the antiangiogenic protein endostatin were raised in men with symptoms of intermittent claudication compared to men who did not have leg pain. This association remained after adjusting for the cardiovascular risk factors of age, diabetes, hypertension, and dyslipidemia. We also adjusted for previous history or treatment of CHD and stroke.

The antiangiogenic effects of endostatin have been of considerable interest to cancer researchers with a number of animal model studies suggesting that endostatin administration inhibits tumour growth [16, 17]. Administration of recombinant endostatin has also been shown to inhibit atherosclerotic plaque neovascularization in a rabbit model [18]. These findings raise the possibility that circulating endostatin could inhibit angiogenesis and arteriogenesis when elevated in peripheral artery disease patients and thereby contribute to the development of lower limb symptoms, such as intermittent claudication.

Despite the demonstrated ability of endostatin to inhibit angiogenesis its overall impact on the outcome of vascular diseases is not yet clear. Studies using a rat model demonstrated that endostatin was upregulated after myocardial infarction and that an antiendostatin antibody promoted mortality [19]. Therefore, in this animal model it is possible that elevated levels of endostatin occurred in response to myocardial injury. Furthermore, downregulation of collagen XVIII, which is cleaved to generate endostatin, has been reported to promote atherosclerosis progression in a mouse model [20]. Of note recombinant endostatin has been reported to inhibit atherosclerosis progression in the same mouse model [21]. Therefore, there are animal data supporting a beneficial effect of endostatin in the context of atherosclerosis.

Human data have been derived from observational studies. Circulating concentrations of endostatin have been found to be raised in patients with a variety of cancers and in some, but not all studies, were associated with poor prognosis [22–25]. Circulating endostatin concentrations have also been reported to be raised in other diseases including inflammatory bowel disease and preeclampsia [26, 27]. Serum endostatin has also been reported to be increased in patients with CHD in some studies, emphasizing the importance of adjusting for this in the current analysis [28, 29]. In the study of Mitsuma and colleagues the level of serum endostatin was inversely related to the extent of coronary collaterals suggesting that endostatin may play a role in inhibiting collateral formation, in keeping with our results [28]. In contrast, in the investigation of Iribarren et al. patients with lower serum endostatin were more at risk of myocardial infarction [29]. There is no current consensus on the role of circulating endostatin in vascular disease; however, the findings of the current study and that of Mitsuma et al. support the hypothesis that circulating endostatin may be a marker of impaired collateral formation or reduced angiogenesis. Further studies on patients with peripheral artery disease are needed to assess this.

A number of possible limitations of this study should be considered including sampling error, measurement error, reverse causality, and residual confounding. While we included over 1000 men in the current study, the prevalence of intermittent claudication was low and the generalizability of the findings to other patients with intermittent claudication, particularly younger patients and females, is not known. The assessment of intermittent claudication was limited to the ECQ. We did not perform other assessments such as measurement of ankle brachial pressure index or arterial imaging. It is therefore possible that we incorrectly allocated patients with peripheral artery disease to the control group. The main aim of our analysis however was to assess the association of endostatin with symptoms of peripheral artery disease which we believe are better captured from the symptom questionnaire than from imaging results. We corrected for confounding factors which we had assessed in our analyses, such as CHD, stroke, and cardiovascular risk factors; however, we were unable to correct for other factors which may have influenced serum endostatin such as cancer. The current study is a cross-sectional observational study; therefore while serum endostatin concentrations were raised in patients with intermittent claudication we cannot draw conclusions as to causality.

In conclusion we report for the first time that serum concentrations of the antiangiogenic protein endostatin are raised in patients with intermittent claudication. Longitudinal studies are needed to assess the temporal association between increases in serum endostatin and occurrence of intermittent claudication using standardized and reliable diagnostic techniques. Further studies are also needed to examine the influence of other potential residual confounding factors that were not measured in this study on the association between endostatin and intermittent claudication, the mechanisms underlying this association, and to clarify whether endostatin, or its modification, plays a role in the outcome of peripheral artery disease.

## Figures and Tables

**Table 1:** Comparison of men with and without intermittent claudication undergoing serum endostatin measurement.

Characteristic	Intermittent claudication	No intermittent claudication	*P* value
Number	106	717	
Age (years)	77.9 ± 4.0	76.7 ± 3.6	0.002
Hypertension	70 (66.0%)	296 (41.3%)	<0.001
Diabetes mellitus	28 (26.4%)	99 (13.8%)	0.001
Dyslipidemia	75 (70.8%)	325 (45.3%)	<0.001
Ever smoker	91 (85.8%)	472 (65.8%)	<0.001
Coronary heart disease	58 (54.7%)	193 (26.9%)	<0.001
Previous stroke	25 (23.6%)	67 (9.3%)	<0.001
Serum endostatin (ng/mL)	145.22 ± 106.93	116.34 ± 66.57	<0.001

Nominal variables are presented as numbers (%) and compared by chi-squared test. Continuous variables are presented as mean (±standard deviation) and compared by Mann-Whitney *U* test.

**Table 2:** Independent risk factors for intermittent claudication in 823 men.

Characteristic	Odds ratio	95% CI	*P* value
Serum endostatin (ng/mL)*			
<93.00	1.00	Reference	
93.00–120.00	2.07	1.15–3.73	0.015
>120.00	1.78	0.98–3.22	0.058
Ever smoking	2.59	1.44–4.67	0.002
Previous stroke	2.12	1.22–3.69	0.008
Dyslipidemia	1.96	1.20–3.20	0.007
Coronary heart disease	1.91	1.20–3.05	0.007
Hypertension	1.77	1.11–2.82	0.016
Age above 76 years^†^	1.67	1.07–2.60	0.023
Diabetes mellitus	1.52	0.90–2.58	0.119

For nominal variables the comparisons are to subjects without the risk factor. *Men with serum endostatin concentrations in the top and middle tertiles were compared with subjects who had serum endostatin in the lowest tertile. ^†^Approximately median value. All factors listed were included in the multivariate model.

## References

[1] Ng EL, Weiland TJ, Jelinek GA, Hadgkiss E, Wilson A Prevalence of and risk factors for peripheral arterial disease in older adults in an Australian emergency department.

[2] Guerchet M, Aboyans V, Mbelesso P (2012). Epidemiology of peripheral artery disease in elder general population of two cities of Central Africa: Bangui and Brazzaville. *European Journal of Vascular and Endovascular Surgery*.

[3] Hirsch AT, Halverson SL, Treat-Jacobson D (2001). The Minnesota Regional Peripheral Arterial Disease Screening Program: toward a definition of community standards of care. *Vascular Medicine*.

[4] McDermott MM, Greenland P, Liu K (2001). Leg symptoms in peripheral arterial disease associated clinical characteristics and functional impairment. *The Journal of the American Medical Association*.

[5] McDermott MM, Fried L, Simonsick E, Ling S, Guralnik JM (2000). Asymptomatic peripheral arterial disease is independently associated with impaired lower extremity functioning: the women’s health and aging study. *Circulation*.

[6] McDermott MM, Carroll TJ, Kibbe M (2013). Proximal superficial femoral artery occlusion, collateral vessels, and walking performance in peripheral artery disease. *JACC Cardiovasc Imaging*.

[7] Seppinen L, Pihlajaniemi T (2011). The multiple functions of collagen XVIII in development and disease. *Matrix Biology*.

[8] Saarela J, Rehn M, Oikarinen A, Autio-Harmainen H, Pihlajaniemi T (1998). The short and long forms of type XVIII collagen show clear tissue specificities in their expression and location in basement membrane zones in humans. *American Journal of Pathology*.

[9] Bao Y, Feng WM, Tang CW, Zheng YY, Gong HB, Hou EG (2012). Endostatin inhibits angiogenesis in hepatocellular carcinoma after transarterial chemoembolization. *Hepatogastroenterology*.

[10] Bai YJ, Huang LZ, Zhou AY, Zhao M, Yu WZ, Li XX (2012). Antiangiogenesis effects of endostatin in retinal neovascularization. *Journal of Ocular Pharmacology and Therapeutics*.

[11] Ling Y, Yang Y, Lu N (2007). Endostar, a novel recombinant human endostatin, exerts antiangiogenic effect via blocking VEGF-induced tyrosine phosphorylation of KDR/Flk-1 of endothelial cells. *Biochemical and Biophysical Research Communications*.

[12] Wu B, Chen H, Shen J, Ye M (2011). Cost-effectiveness of adding rh-endostatin to first-line chemotherapy in patients with advanced non-small-cell lung cancer in China. *Clinical Therapeutics*.

[13] Norman PE, Flicker L, Almeida OP, Hankey GJ, Hyde Z, Jamrozik K (2009). Cohort profile: the health in men study (HIMS). *International Journal of Epidemiology*.

[14] Iribarren C, Herrinton LJ, Darbinian JA (2006). Does the association between serum endostatin, an endogenous anti-angiogenic protein, and acute myocardial infarction differ by race?. *Vascular Medicine*.

[15] Yeap BB, Alfonso H, Chubb SA Lower plasma testosterone or dihydrotestosterone, but not estradiol, is associated with symptoms of intermittent claudication in older men. *Clinical Endocrinology*.

[16] Sun J, Deng L, Duan Y (2012). Inhibitory effect of endostatin combined with paclitaxel-cisplatin on breast cancer in xenograft-bearing mice. *Experimental and Therapeutic Medicine*.

[17] Braga MDS, Chaves KB, Chammas R, Schor N, Bellini MH (2012). Endostatin neoadjuvant gene therapy extends survival in an orthotopic metastatic mouse model of renal cell carcinoma. *Biomedicine and Pharmacotherapy*.

[18] Mao W, Kong J, Dai J (2010). Evaluation of recombinant endostatin in the treatment of atherosclerotic plaques and neovascularization in rabbits. *Journal of Zhejiang University B*.

[19] Isobe K, Kuba K, Maejima Y, Suzuki J-I, Kubota S, Isobe M (2010). Inhibition of endostatin/collagen XVIII deteriorates left ventricular remodeling and heart failure in rat myocardial infarction model. *Circulation Journal*.

[20] Moulton KS, Olsen BR, Sonn S, Fukai N, Zurakowski D, Zeng X (2004). Loss of collagen XVIII enhances neovascularization and vascular permeability in atherosclerosis. *Circulation*.

[21] Moulton KS, Heller E, Konerding MA, Flynn E, Palinski W, Folkman J (1999). Angiogenesis inhibitors endostatin or TNP-470 reduce intimal neovascularization and plaque growth in apolipoprotein E-deficient mice. *Circulation*.

[22] Li M, Liu F, Sun P (2012). Correlations between serum levels of vascular endothelial growth factor and endostatin with clinical pathological characteristics of patients with gastrointestinal cancers. *Hepatogastroenterology*.

[23] Mo HY, Luo DH, Qiu HZ (2013). Elevated serum endostatin levels are associated with poor survival in patients with advanced-stage nasopharyngeal carcinoma. *Clinical Oncology*.

[24] Szarvas T, László V, Vom Dorp F (2012). Serum endostatin levels correlate with enhanced extracellular matrix degradation and poor patients’ prognosis in bladder cancer. *International Journal of Cancer*.

[25] Aref S, El-Sherbiny M, Azmy E (2008). Elevated serum endostatin levels are associated with favorable outcome in acute myeloid leukemia. *Hematology*.

[26] Oikonomou KA, Kapsoritakis AN, Kapsoritaki AI (2011). Angiogenin, angiopoietin-1, angiopoietin-2, and endostatin serum levels in inflammatory bowel disease. *Inflammatory Bowel Diseases*.

[27] Wathén K-A, Ylikorkala O, Andersson S, Alfthan H, Stenman U-H, Vuorela P (2009). Maternal serum endostatin at gestational weeks 16-20 is elevated in subsequent pre-eclampsia but not in intrauterine growth retardation. *Acta Obstetricia et Gynecologica Scandinavica*.

[28] Mitsuma W, Kodama M, Hanawa H (2007). Serum endostatin in the coronary circulation of patients with coronary heart disease and its relation to coronary collateral formation. *American Journal of Cardiology*.

[29] Iribarren C, Herrinton LJ, Darbinian JA (2006). Does the association between serum endostatin, an endogenous anti-angiogenic protein, and acute myocardial infarction differ by race?. *Vascular Medicine*.

